# On the use of evolutionary mismatch theories in debating human prosociality

**DOI:** 10.1007/s11019-021-10025-4

**Published:** 2021-05-12

**Authors:** Andrés Segovia-Cuéllar, Lorenzo Del Savio

**Affiliations:** Ludwig-Maximilians-Universität München, Munich, Germany

**Keywords:** Prosociality, Enhancement, Human Nature, Evolution, Cooperation

## Abstract

According to some evolutionary theorists human prosocial dispositions emerged in a context of inter-group competition and violence that made our psychology parochially prosocial, ie. cooperative towards in-groups and competitive towards strangers. This evolutionary hypothesis is sometimes employed in bioethical debates to argue that human nature and contemporary environments, and especially large-scale societies, are *mismatched*. In this article we caution against the use of mismatch theories in moral philosophy in general and discuss empirical evidence that puts into question mismatch theories based on parochial prosociality. Evolutionary mismatch theories play at best a rhetorical role in these moral debates and may misrepresent the status of relevant evolutionary research. We finally recommend that moral philosophers interested in the evolutionary literature also engage with dispositions such as xenophilia and social tolerance to counterbalance the focus on psychological mismatches adopted so far.

## Introduction

Research on the origins of human moral dispositions is sometimes employed in moral philosophy and bioethics. The debate on moral enhancement exemplifies one way such research can be employed there. Moral enhancement is the proposal to improve the moral psychology of the human species, if necessary by biomedical means. What aspects of moral psychology, if any, need improvement? Some theorists rely on research on the origins of human cognitive and social dispositions to answer this question. They do so to identify and describe those psychological limitations that may make the resolution of a host of contemporary issues intractable, from global collective action problems to the threats associated with the misuse of modern technologies (Persson and Savulescu 2014, 2017, 2019; Powell and Buchanan 2016). This focus on human origins is sometimes undertheorized. It is however *not* a trivial focus, not only because inferences regarding human evolution and current psychological dispositions are an intricate matter, as the long-standing debate on evolutionary psychology testifies (Kitcher 1985; Gray et al. 2014; Buller 2005; Dupré 2001; Mameli 2012), but also because what ultimately matters in moral debates is the forward-looking questions whether certain moral dispositions are undesirable and whether and how such dispositions can be changed, rather than the backward-looking question of whence they come.

The connection between these different sort of questions about moral psychology must be clarified in order for moral origins to become relevant in such discussions.

Alas, this connection is often left out and hence we would like to critically reflect on the inferences that may support the focus on moral origins. We conjecture that, once made explicit, the connection between backward-looking questions regarding moral psychology and the justification of moral enhancement would consist of two main assumptions:

1. Reconstructions of the human deep past bring into relief differences between the ancient environments in which the bulk of human psychological dispositions evolved and contemporary socio-ecological environments, thereby allowing the identification of *mismatches* between evolved psychological dispositions and the latter environments;
2. Evolved traits can only change in the long-run, with the *slow pace* of biological evolutionary change, thus posing hard constraints on the short-and middle-term horizons of human agency, i.e. in policy-making and politics.

Several psychological dispositions purportedly fit this template of *slowly changing, mismatched dispositions* that frustrate social, moral and political progress: parochial prosociality (i.e. the tendency to preferentially cooperate with in-groups and hence to preferentially exclude, discriminate against and assault strangers), nepotism, biases towards the near future, emotions involved in ascriptions of personal responsibility, etc. Persson and Savulescu have even coined a phrase that explicitly plays with the common-sense and evolutionary meanings of *fitness*: humans may be a species that is “*unfit* for the future” (Persson and Savulescu 2014, italics added).

In a series of papers and a book, Buchanan and Powell have done much to explain the difficulties involved in inferences based on human origins for questions pertaining to how societies can and cannot be organised, and what can be done to ameliorate human dispositions that are purportedly deficient (Buchanan and Powell 2015, 2016, 2018; Powell and Buchanan 2016). They have done so by emphasising the ubiquity of *plasticity* in the human psychological and behavioural repertoire, i.e. the conditional expression of certain responses to different environmental stimuli, and hence the ample leeway that tinkering with environmental conditions may provide to those seeking moral change. In so doing, they criticise both what they call “bioconservatives”, who believe that evolved human dispositions pose hard constraints on social change and “bioliberals”, who also believe that evolved dispositions are hard constraints but argue that biomedical interventions may—and to the extent they can, should—address such limitations by enhancing human psychology. In other words, Buchanan and Powell carefully qualify assumption (2) as we describe it above: there may be evil evolved dispositions haunting human nature and hence our hope for moral reform but they do not necessarily change slowly, as they are more plastic than other theorists (such as the aforementioned Persson and Savulescu) believe.

In this article we take a step forward in the critical assessment of recent uses of research on human origins in practical ethics by critically discussing assumption (1), the mismatch assumption, and criticising how Buchanan and Powell conceptualise plasticity. We focus both on evolutionary mismatch theories in general and on one specific theory on which some bioethicists have built. This is the theory that many social dispositions, including the emotional repertoire that underpins what is usually called “morality”, evolved in a socio-ecological environment characterised by small cooperative groups warring between each other and competing for resources. Bioconservatives, bioliberals, and even their critics Buchanan and Powell share such a narrative about human moral origins. We argue that this narrative is more controversial than all these theorists make it out to be, and that it is theoretically unsatisfying to the extent that it rests on assumption (1). Buchanan and Powell argue that, despite psychological plasticity, human moral origins make large-scale, inclusive societies, and the forms of cooperation that are endemic therewithin, an unstable evolutionary anomaly. While the instability of any social order may be one of the more significant lessons of evolutionary approaches to social and political phenomena (Bagg 2019), we take issue with the language of inclusivity and large-scale cooperation being *anomalous*, which is an ontogenetically sophisticated re-statement of a mismatch theory of human nature. We take issue, in other terms, even with the more sophisticated versions of mismatch theorising as represented by the recent works of Buchanan and Powell.^1^

We argue that: human extreme behavioural and psychological flexibility lends itself to cherry-picking aspects of our evolved nature that fits pre-established normative agendas. This applies to the formulation of mismatch theories as well, which are often post-hoc reconstructions of moral theories in evolutionary terms (Sect. 2); the image of a Upper-Pleistocenic life haunted by inter-group violence encounters several empirical and theoretical problems, and it is not scientific common sense, but a hotly debated area of empirical disagreement (Sect. 3); a complementary focus on the better sides of our nature is also viable, i.e. sympathetic curiosity towards outgroups, care of distant others and joint production with strangers (Sect. 4); to conclude, we recommend moral philosophers engage with recent research regarding tolerance, intergroup cooperation and generosity: such an engagement would complement the unilateral emphasis on human limitations of many theorists—a unilateral emphasis due to their reliance on evolutionary mismatch theories (Sect. 5). Studying human origins can enlighten these further sides of human nature if only we let go of the one-sided picture of slowly changing, mismatched evolved dispositions that obfuscate our view of the human past and hence the present. Inferences between research on human origins and the justification of human enhancement should be made cautiously: such research are mainly a good source, along others, for enriching our understanding of moral capacities.

## Mismatch theories as moral and political theories

The employment of evolutionary mismatch theories in moral and political philosophy is not new.^2^ More than 40 years ago, in the epilogue of his work on “the constitution of freedom”, the economist Friedrich Hayek claimed that “the conduct required for the preservation of a small band of hunters and gatherers, and that presupposed by an open society based on exchange, are very different” (Hayek 1979, p. 164). Characteristically, he believed that large-scale societies can only be organised under the *“*guidance of the coordination of a far-ranging division of labour by variable market prices”: in brief, market institutions (ibid.). Markets are in turn sustained by the diffusion of “certain gradually evolved moral beliefs”, which result from an unconscious cultural evolutionary process pitting human societies against each other. This evolutionary process spontaneously produced allegedly superior forms of social cooperation, including large-scale “open societies” (Luban 2019). Hayek warned that such evolved moral beliefs are transient and hence that large-scale societies are fragile, a fragility that was purportedly most evident in his lifetime:

At present, however, an ever increasing part of the population of the Western World grow up as members of large organizations and thus as strangers to those rules of the market which have made the great open society possible. [...] In consequence, the long-submerged innate instincts have again surged to the top. Their demand for a just distribution in which organized power is to be used to allocate to each what he deserves, is thus strictly an atavism, based on primordial emotions (ibid. p. 165).

This Hayekian theory is a paradigmatic case of a mismatch theory. *Modern* large-scale societies can only be organised through market institutions but unfortunately a *primordial* sense of justice in allocation threatens such institutions, with pending disastrous consequences. The bottom line of any mismatch theory is that human psychology is riddled with what Hayek calls “atavisms”—in this case: intuitions about deserts that allegedly fit hunter-gatherer cooperative needs—whose re-emergence endangers more modern and more efficient, forms of cooperation. The threat that Hayek has in mind is overtly political: the capture of the coercive capabilities of the state (“organised power”) by “members of large organisations”, i.e. interest groups such as trade unions, which may interfere with the allocation of resources based on unfettered market prices. Plainly, Hayek’s mismatch theory is a reformulation in evolutionary disguise of his central neoliberal theories.

We are not interested here in whether or not these Hayekian ideas are defensible. Hayek’s theory is useful because it illustrates the extent to which normative preferences—bluntly put: a political programme—orientate practical theorising purportedly based on evolutionary considerations. There is no mismatch between a *Paleolithic mind* (Barcow et al. 1996), neutrally described, and modern conditions, equally neutrally described. The Hayekian mismatch holds between certain alleged human dispositions that the theorist loathes (specifically: a sense of a just desert in allocation) and those features of contemporary societies that he intends to defend and expand (namely, market institutions). In this Hayekian excerpt, the discussion of neoliberal ideas in terms of hunter-gatherer morality versus open societies is little less than a rhetorical spin. To be sure, there are examples of more in-depth engagement with evolutionary anthropology both in Hayek himself^3^ and other scholars. But this excerpt is no isolated, exceptionally unconvincing example of a mismatch theory. This is because the dazzling flexibility of human psychology and behaviour allows for the picking and choosing as maladaptive those psychological dispositions that fit pre-established normative agendas. In fact, such a normative agenda is *necessary* to decide which dispositions matter, and what features of modern societies—also very variable indeed—need to be preserved from purported atavisms. We hasten to note that this normative character of mismatch theories is no damning objection. Yet it is important to keep in mind that the language of *constraints* and *limitations* that accompanies evolutionary mismatch theories is *not* descriptive language. It is normative language, where the moral and political programme of the theorist leads their evolutionary musings, rather than vice versa.

Claims that evolved dispositions constrain social arrangements should be considered cautiously for another reason that does not have to do with the dazzling variety of human dispositions but—on the contrary—with the factors that constrain such variety (see also Sterelny 2018). Mismatch theories pitch slowly changing psychological dispositions against rapidly changing human norms and institutions. This apparently trivial view, which Hayek also espoused, is misleading insofar as it constructs the psychological side and the institutional side as if the former could not be influenced by the latter, neither evolutionarily or developmentally. But the observed variability of human social dispositions is constrained by extant norms and institutions as much as the variability of the former is constrained by the latter. This is because human dispositions do not develop—and hence evolve—in a vacuum, but in a social niche made up of social norms and institutions. The variety of psychological dispositions that we observe is, among other things, a function of the norms and institutions that actually exist (Muthukrishna 2021). Mismatch theories, in other terms, are based on a simplistic view of the relationship between evolved psychological dispositions on the one hand, and the evolution of norms and social institutions on the other. Speculations on what psychological variability would look like were norms and institutions different are not impossible; but they are bound to be highly tentative, and for this reason bound to be influenced by normative preferences.

Persson and Savulescu (2017) write that “The present time brings existential threats which human moral psychology, with its cognitive and moral *limitations and biases*, is unfit to address” (p. 286, italics added). They continue: “We have hypothesized that these limitations are the result of the evolutionary function of morality being to maximize the fitness of small cooperative groups competing for resources” (ibid.). The redundancy of the latter evolutionary hypothesis stands out in this quotation: the identification of moral limitations is evidently done on independent grounds, which they outline so: “Exponentially increasing, widely accessible technological advance and rapid globalization create threats of intentional misuse (e.g. biological or nuclear terrorism) and global collective action problems, such as the economic inequality between developed and developing countries and anthropogenic climate change, which human psychology is not set up to address” (ibid.). None of this depends on evolutionary considerations. These perspectives are based on visions of what humans should become, rather than theories of what they and their ancestors have been (i.e. backward-looking questions about the origins of moral psychology). The latter serves the purpose of lending credibility to claims that some human dispositions are recalcitrant to change, via the dubious assumption that what has “evolved” must change slowly.

If a normative agenda rather than evolutionary theory drives the identification of relevant problematic dispositions, appeals to evolutionary theory are bound to be highly selective and to fit pre-existent normative projects. We do not want, however, to claim that evolutionary research is irrelevant here. Whether or not such evolutionary research is informative in such discussions cannot be decided a priori and, moreover, normative preferences are as good a starting point as any for good empirical research. In fact, we also rely on evolutionary research to show that mismatch theories based on “small cooperative groups competing for resources” are more controversial than their proponents make them out to be, and informed by patent normative preferences. Our viewpoint is equally value-laden: we do not intend to advance another viewpoint as if ours were a neutral, value-free assessment of these matters. We rather suggest that other perspectives are equally compelling, and that they expand our sense of possibility and hence—potentially—our instruments for change.

We also do not want to imply that mismatch theories have no theoretical use whatsoever. The general idea of an “evolutionary mismatch” follows from the Darwinian lesson that biological adaptations should be understood in the context of the historical environment that gave rise to them. This important lesson has contributed to the abandonment of teleological views of organisms as functioning whole down to their smallest components, and hence of the natural state of organisms as a normative guide to how organisms should be. This is fundamental.^4^ But aside of this critical role, the pervasive adoption of an evolutionary mismatch standpoint runs the risk of reintroducing from the backdoor fallacious inferences regarding how organisms *should be* based on how *they are,* to the extet they lend a seemingly descriptive language of “maladaptness” to moral discussions*.*

## Inclusivism as a luxury good: first problems

“We don’t just belong to human kinds; we prefer our own kind and we’re easily persuaded to take against outsiders” (p31) says the moral philosopher Appiah (2018) on the parochial nature of human sociality. Ideals of inclusion—of migrants, minorities and other oppressed groups—play a prominent role in contemporary liberal societies. It is hence no surprise that psychological dispositions to exclude out-groups, to refuse cooperation, or to do them harm are the focus of much engagement of moral philosophers with psychological research. The scholarly interest in parochial prosociality arguably peaks whenever conflicts that can be seen as driven by exclusivist tendencies spread: ethnic hatred and genocide two decades ago, anti-immigration xenophobia today.

The “groupish” tendencies of the human mind are a long-standing research topic of social psychology (Billig and Tajfel 1973). People tend to sympathize and rate the members of their group as more likable and trustworthy and tend to cooperate with in-group partners even if their assignment to groups follows an arbitrary decision (Brewer 2010). More recently, these tendencies have become the object of an expanding programme in evolutionary anthropology and psychology (e.g. Richerson and Henrich 2012; Haidt 2013). This engagement of evolutionary theorists is largely due to the rehabilitation of group selection models (Sober and Wilson 1998). The central idea is that the outstanding prosocial tendencies of modern humans, apparently disadvantageous for any individual, may have evolved in contexts where it paid off to selectively cooperate with in-groups because groups are under selective pressure *as groups*: and groups of cooperators do better than others.^5^ Modelling work shows that this is possible under certain restrictive conditions that are thought to be rare in nature, but possibly common in human evolutionary history. The first condition is the presence of sharp intergroup competition. Evidence of a bellicose human past—our main critical focus below—would ensure that this condition is met. The second condition is strong homogeneity in terms of cooperative dispositions within groups and its correlate, namely intergroup variability with respect to cooperative dispositions. This second condition may have been satisfied in human evolutionary history because of human cognitive sophistication. Such sophistication, for instance, facilitates the emergence of punishment mechanisms against cooperative non-conformity, which in turn magnifies intergroup variability (Boyd and Richerson 1992; Bowles and Gintis 2011). These group selectionist models suggest that prosocial and groupish dispositions may be coupled, which would explain why the kinds to which humans belong—no matter how arbitrary—fine-tune the propensity to cooperate with others, as in the quote above by Appiah (Choi and Bowles 2007). An Upper-Pleistocenic scenario where competition between small groups was widespread is instrumental for these theories of the evolution of parochial prosociality.

This body of evolutionary theory is taken to bear on recent debates in bioethics by Buchanan and Powell: “Data and theory suggest that morality evolved in a highly competitive intergroup environment in which extending altruism to out-group members would often have been evolutionarily deleterious. The result was the evolution of a highly parochial altruism” (Buchanan and Powell 2018, p. 79). As a result, they write: “Parochial altruism, which consists in the combination of in-group favoritism/empathy and out-group antagonism/antipathy, is among the most cross-culturally robust features of human moral psychology and a direct prediction of group selectionist accounts of morality” (Buchanan and Powell 2018, p. 130).

Both Buchanan and Powell and those whom they criticise agree on such a perspective on human origins and nature. But they disagree with regards to the practical consequences of this purported scientific consensus. Bioconservatives pedal such facts to argue that the perspective of inclusive, open societies is bleak as they run against the grain of deep-seated human dispositions towards xenophobia. Bioliberals agree with the latter’s pessimistic assessment but suggest that hope may come from a mixture of biomedical interventions—the majority of which do not exist yet—and a range of further behavioural measures. Beyond their disagreements, however, all these theorists espouse a mismatch concept of human nature: parochial sociality may have been cooperatively beneficial in the past when small human groups were competing, but became a problem once large-scale modern societies emerged. In modern societies inclusive moralities, non-strategic cooperation and cooperation with perceived out-groups are widespread and, arguably, desirable.

What distinguishes Buchanan and Powell’s account from bioliberals and bioconservatives is chiefly the sophistication of the former with regards to the ontogenesis and nature of parochial prosociality (especially the lengthy treatment of the issue in Buchanan and Powell 2018). They speculate that, in certain conditions, cooperative intergroup encounters may have paid off despite intense intergroup competition in Upper-Pleistocenic life. This is because people did benefit from intergroup trade, mating and other exchanges even if the default status of intergroup relationships was war. If so, they reason, it would make sense to have *conditionally* parochial social tendencies, i.e. dispositions that are able to switch on and off according to the appropriate circumstances. Buchanan and Powell compare such flexibility to a textbook model of phenotypic plasticity: the conditionally expressed defensive armour (a thorny coat) that certain water fleas (*Daphnia sp*) develop when exposed to chemical stimuli associated with predators. Conditional parochial plasticity, they speculate, could similarly be an *adaptive* mechanism of human psychology. Buchanan and Powell further hypothesise that triggers of parochial behaviour—analogous of the chemical stimuli associated with predators in *Daphnia*—include the perception of intergroup competition in conditions of scarcity and the threat of contagious diseases. The stability of large-scale societies based on widespread cooperation between *strangers* (Seabright 2006) is, in this perspective, a luxury good that becomes available in times of plenty, when there is no scarcity and no epidemics are in sight. Unless such plentiful conditions are established, however, any large-scale society is threatened by the re-emergence of the atavic groupish tendencies of the human mind. In this theory, there is a mismatch (as per assumption 1), but psychological dispositions can change not only in the long run of biological evolution—which would be next to irrelevant for actions—but in the more rapid times of ontogenesis (contrary to assumption 2).

This is a sophisticated theory if compared to blunt claims that parochial sociality is recalcitrant to intervention because it is allegedly part of our evolved nature. Yet many objections could be nonetheless raised, partially because of this sophistication, which makes the theory empirically more vulnerable. Recent cross-cultural research has put into question the relevance of (the perception of) competition and the risk of infectious diseases as triggers of parochially prosocial attitudes (Romano et al. 2017). Claims that the plasticity of parochial prosociality was *adaptive* are merely hypothetical in the absence of further empirical and theoretical work on the fitness advantages that it could provide in ancestral environments. The analogy with the water flea armour may be misleading to the extent it suggests a switch-like system of behavioural flexibility, whereas such flexibility may be, in humans, under cognitive control.^6^ Importantly, no sufficient attention is given to the presence of different psychological profiles in human populations (see Romano et al. 2017 for its importance when discussing parochial prosociality). And, finally, it has been suggested that it is mechanistically implausible that tendencies for taking against outsiders and tendencies for cooperation are developmentally tightly connected at a biological level (Sterelny 2016).

The fact that in-group attachment and outgroup hostility are part of the same phenomenon was initially proposed by Sumner (1906/2007). However, recent scientific evidence has challenged this idea (Brewer 1999). Corr et al.(2015) and Yamagishi and Mifune (2016) found no evidence of a correlation between in-group cooperation and out-group hostility, and Sapolsky has claimed that in-group biases can be interpreted biologically without appealing a fixed orientation for inter-group competition (2017). Human children tend to over-imitate (Horner and Whiten 2005), tend to synchronize and conform their behaviors with others (Haun et al. 2014), are more sensitive to the communicative and linguistic signals of their groups (Kinzler et al. 2011) and show more sympathy to in-group partners (Rhodes and Chalik 2013). Moreover, children use to exclude out-group members, tend to present their best image to peer groups, and prefer members of their social groups across a variety of social situations (Killen et al. 2013; Killen et al. 2015). However, children do not automatically dislike out-group members, and their affiliation depends on social knowledge and their social-cognitive abilities (Rutland and Killen 2015).

There is no doubt that Buchanan and Powell could provide an even more sophisticated theory that overcomes such difficulties. We want however to take issue with the general narrative that they share with other theorists. This is the narrative that emphasises between-group competition in the evolution of the emotional repertoire that underpins human sociality and morality. The data and associated theories are much more open-ended with regards to the evolution of parochial sociality than all these theorists suggest: they seem to be working with a received view where there is none available. As we have argued on mismatch theories in general, the mismatch theory of parochial prosociality is rather forward-looking than backward-looking. We suspect that the theory is based on the pre-eminence—which we entirely endorse—of inclusivist ideals in recent political thought, and on the social diagnosis that deep-seated xenophobic tendencies may be driving the current nationalist moment in several countries. We would like to repeat that this genealogy would not be a major theoretical flaw, *unless* narratives of a bellicose past were presented as scientific consensus, thus preventing the exploration of alternative perspectives on the evolution and the future of human sociality.

## A bellicose species?

A long-standing line of scholarship has repeatedly warned against the aggressive and xenophobic dispositions of the human species. This could be called, after Lee (2018), the “bellicose” tradition of reflections on human nature (e.g. Wrangham and Peterson 1997; Pinker 2011). Such a tradition, in its modern form, chiefly relies on the claim that the common ancestors of apes were prone to aggressive behaviours towards out-groups, and the claim that hunter-gatherer Upper-Pleistocene life was characterised by continuous intergroup wars. The first claim depends on reports of violence towards out-groups among chimpanzees and the thesis that the latter is a good baseline for the ancestral ape condition. The second claim depends on reports of violence towards out-groups in the ethnological record and the thesis that the current hunter-gatherers’ ways of life can be extrapolated, albeit cautiously, to Upper-Pleistocenic humans. Of all these theses and reports, only the first is relatively uncontroversial^7^; the others are the object of heated empirical, theoretical and political debate (Lee 2018; see also Hames 2019).

The hypothesis that our prosocial dispositions were forged in an environment characterised by epidemic conflict between small-scale bands of foragers, depicting a competitive Upper-Pleistocenic human life, continues the bellicose tradition. This picture of ancestral foraging conditions is, however, much debated. Life in the Pleistocene was certainly no paradise. Interpersonal violence was possibly very common. And intergroup violence does belong to the spectrum of behaviours that are documented in foraging societies for which we have ethnographic reports (Kelly 1995). But the archaeological record lacks evidence of widespread intergroup violence for the period. Moreover, intergroup violence may have paid off at a later stage only, once concentrated resources appeared up for grabs, i.e. after the advent of pastoralism and sedentary agriculture (Sterelny 2016). Importantly, war-like non-state human groups that are often mentioned in the bellicose tradition as evidence of conflictual ancestral conditions^8^ are decisively *not* hunter-gatherers, and hence do not belong to the spectrum of behaviours that humans exhibited in the Upper-Pleistocene.^9^

In fact, the very assumption that, in the Upper-Pleistocene, foraging groups competing for resources were universally *small* is controversial. While the hypothesis that foraging life was organised around fission–fusion bands that relied on a network of ca. 150 individuals has profoundly influenced recent research on social evolution (Dunbar 1993, 2010), there is evidence that some extant hunter-gatherers are organised in multilevel groups comprising 10X more individuals (Bird et al. 2019). This scale is not dissimilar from that surrounding a typical member of agricultural and industrial societies. If so, claims that social organisation in the Upper-Pleistocene comprised “small-bands” are also to be taken cautiously, as one of the traditional points of evidence in its favour—the ethnological record—may be less conclusive than previously thought.

Obviously, none of these controversies is a *decisive* argument against the bellicose view of human nature. Yet it is very important to remark that more hypotheses are available pertaining to the emergence of prosocial parochiality than is usually discussed in these debates. Buchanan and Powell are interested in the interplay between the adaptive components of moral psychology and the cultural evolution of moral norms. This is indeed an important focus in reflections about human nature, which does away with the simplistic assumption (2) according to which evolved dispositions only change slowly. Yet such an interplay may also be investigated with reference to later, Holocenic cultural dynamics, where indeed evidence of widespread intergroup conflict is prominent (Sterelny 2016). *Cultural* group selection may have led to the emergence of a set of beliefs, norms and institutions that magnified and coupled the pre-existing groupish and cooperative dispositions of the human mind. Such a cooperative function is documented for religions and imperial ideologies, which often deepened within-group cooperation at the expenses of cooperative outreach, thereby promoting a conflictual attitude towards, respectively, infidels and barbarians (e.g. Wilson 2002; Turchin 2015). Notably, such a cultural origin, if confirmed, would not alone prove that parochial prosociality is easily malleable. This is because parochial prosociality may be entrenched in the psychological repertoire of human beings as a result of slowly changing cultural factors rather than slowly changing biological factors. Cultural traits may have surprising endurance too. The deepest past and biology do not have any particular evidential priority in the question of whence a disposition comes from and whether it can be changed. Mismatch theories misleadingly restrict such a focus to biological factors narrowly conceived. But the ontogenetic impact of moral codes, social norms and laws should be taken as seriously as biological factors in such debates.

We propose to take a broader perspective. Human ultrasociality, our capacity to cooperate in very large groups largely comprised of non-kin and strangers, is not anomalous because it runs against evolved dispositions. Yet it is rather unique in nature. Recent research on the emergence of human ultrasociality looks at both what makes humans so distinctively unique, and especially their capacity for cumulative cultural evolution (Henrich 2015), and the preexisting dispositions that may have paved the way for the emergence of large-scale societies—dispositions that do exist in other species as well. Researchers studying the former concentrate on processes such as: the adoption of universalist moral codes, the spread of organised religions, and the birth of impersonal institutions such as markets and states. These are all evolved cultural technologies that made ultrasociality possible, and they are also relevant in discussions regarding the origin and stability of inclusive sociality. We leave this topic for another time and focus on the second, complementary research branch, namely the motivational and emotional aspects of sociality.

Against the grain of the bellicose view, it can be hypothesised that inclusivist, peaceful dispositions may also be ancient, and that they may have been, moreover, evolutionary consequential in moulding the human species. There is accumulating evidence that human beings underwent a process of self-domestication that resulted in an exceptionally tolerant psychological profile (Wrangham 2019; Hare 2017; Cieri et al. 2014). Humans display physiological, morphological and behavioural features that are typical of domesticated animals and a comparatively socially tolerant ape such as the bonobo.^10^ At the behavioural level, humans have a high sensitivity to social clues, powerful cooperative communication and low aggressivity towards each other.

In humans as well as in bonobos, such reduced aggressivity extends to out-groups. What could have led to such psychological profiling is a matter of hypothesis, but it is suggestive that coalitional behaviours against dominating males may have been the leading driver in both humans and bonobo, albeit in different forms and time frames (Wrangham 2019). Alternatively, the trait may be ancestral in apes (for this debate, see Hare and Wrangham 2017; Gonzalez-Cabrera 2017), and due to evolutionary processes further back in time (Raghanti et al. 2018). A further hypothesis is that complex forms of human social cooperation could be the result of the convergence of enhanced social cognitive skills and unique prosocial motivations emerging in the developmental niche of alloparental care, i.e. the care of newborns and infants by an extended group of individuals (Burkart et al. 2009; Burkart et al. 2014; Burkart & Van Schaik, 2016). A relation between alloparental care and enhanced prosociality is well established in comparative studies, including in several species of primates (Burkart et al., 2014), and has been found with other species such as corvids (Horn et al. 2016).

This body of theories is still in rapid development: we do not intend to rely on it to advance particular claims about human psychological dispositions. But it is important in this context to point out the unnecessary limitations that are imposed on such discussions by a unilateral focus on violence, exclusivity and traits that are undesirable. Pisor and Surbeck (2018) have claimed that much of the work in evolutionary anthropology regarding the peaceful or conflictive nature of human species was unduly focused on environmental conditions that favour or promote intergroup conflict. Much less studied are instances of ecological pressures that favour tolerant intergroup encounters and disincentive aggression. Along group selection models, they argue, there is a need to “theorize on individual-level selection pressures that may favor intergroup tolerant encounters” (ibid.). Enhanced benefits in the transfer of goods, mating and food acquisition, as well as social learning, may incentivise tolerance towards out-groups in humans. In particular, spatial and temporal fluctuations in resource availability could have favoured tolerant relations between communities or human individuals living in close spatial proximity. Mutual aid, rather than war, could be the evolutionary offspring of scarcity.

Importantly, if this is correct, the resulting psychological profiles of humans cannot be usefully represented by deterministic models of adaptive plasticity such as *Daphnia*. Humans are ambiguous and paradoxical through and through. They are also highly cognitively sophisticated. With regard to violence towards outgroups, Boehm has thus summarised such a body of hypotheses: “Although hunter-gatherers are able to resolve conflicts preemptively, they also use mechanisms, such as truces and peace pacts, to mitigate conflict when the costs become too high. Today, humans retain the genetic underpinnings of both conflict and conflict management; thus, we retain the potential for both war and peace” (Boehm, 2012, p. 844). If plasticity allows for such sophisticated strategising, however, we doubt that, in order to dampen parochially prosocial tendencies, ensuring that a perception of plenty and lack of epidemic disease is neither necessary nor sufficient. The messier realm of politics, of who does what to whom, cannot be bypassed by focusing on a narrow selection of psychological dispositions. The focus needs to be broad and to thematise how the host of conflicting human psychological tendencies are mobilised in current political circumstances.

## Conclusions

Theories of human enahancement have made considerable advances in discussing the import of evolutionary anthropology for debates regarding how robust psychological dispositions may frustrate important human projects, including addressing lethal threats for the human species. We have argued, however, that such debates may be hampered by their widespread reliance on evolutionary mismatch theories. Such theories are, very often, normative theories in disguise, and they conceptualise the relationship between biological and cultural evolution in a simplistic manner. Rather than abandoning the naturalistic perspective that proponents of mismatch theories advance, we recommend looking at research that questions the narrative of a bellicose human past, and a complementary focus on embracing the better sides of our nature: xenophilia, inclusivity and intergroup prosociality. These are also part of the emotional repertoire that constitutes human morality. With regards to parochial prosociality, we suggest that a focus on the cultural evolution of moral codes, religion and the like can be fruitful if the aim is to understand the interplay of psychology and moral norms.

It was not our intention, here, to systematically review the immense body of literature on bellicose theories of human nature and recent group selectionist models of social evolution. With our critical observations, we want to emphasise that there is no received view of social evolution that can be uncontroversially used in moral debates. This is not a case—if indeed there are any—where an established piece of scientific knowledge can be transferred in discussions of practical ethics. The openness research in evolutionary anthropology regarding parochial prosociality is no reason, however, to abandon the field and return, as it were, when a consensus emerges (if it ever does). Rather, other perspectives may be equally relevant for the pressing questions asked in these debates.

We finally call for a change of emphasis in moral debates relying on evolutionary research. We should not only look for atavisms and ask whether these make human nature wicked, vulnerable or inadequate. Rather, we should ask how evolved human dispositions can be transformed and mobilised for change, to fulfil projects that we deem desirable. Humans are, no doubt, an exclusivistic and bellicose species. But we also have the unprecedented capacity to help distant others, to jointly produce with strangers, and to exchange genes and cultures across group boundaries. We may need to do all of these.

## Footnotes

1 Other articles with a similar target but a different account of moral plasticity are Lewens (2019) and Sterelny (2019).

2 See also Milam (2019) for discussions of the reception of evolutionary anthropology in public debates in the post-war United States: the diffusion of mismatch theories reaches epidemic proportions in evolutionary anthropology targeting non-specialists.

3 On the engagement of Hayek with evolutionary theory, see Beck (2018).

4 Equally fundamental are Darwinian ideas regarding gradualism, i.e. the slow pace of evolutionary change: but while gradualism once had a critical role in dismantling non-evolutionary world-views, it is now known to be partially false, as many traits can change more rapidly than Darwin thought possible.

5 The basics of these ideas had been already sketched in a much-quoted Darwinian paragraph: “There can be no doubt that a tribe including many members who, from possessing in a high degree the spirit of patriotism, fidelity, obedience, courage, and sympathy, were always ready to give aid to each other and to sacrifice themselves for the common good, would be victorious over most other tribes; and this would be natural selection. At all times throughout the world tribes have supplanted other tribes; and as morality is one element in their success, the standard of morality and the number of well-endowed men will thus everywhere tend to rise and increase.” (Darwin 1871, p. 166).

6 No model is a replica, but this model may fail exactly on the point being discussed, i.e. the speed of change of certain dispositions. Development may be faster than evolution, but it is still slower than cognitive control on behaviour.

7 It has been argued that intergroup aggression in chimpanzees may be a result of anthropogenic habitat disruption. This thesis is also controversial, though to a lesser degree.

8 Even these reports have been criticised, either as excessively selective or as artefacts of Western colonial entrenchment and, therefore, profound ecological deterioration.

9 One source of confusion here is the conflation of several different types of social groups in the category of non-state societies. While this may be useful for the purpose of discussing the merits and demerits of states, it is a category that is irrelevant in discussion of Upper-Pleistocenic lifeways. There are non-state societies among horticulturalist and agriculturalist as well, and in fact these were generally quite common for millennia after the emergence of agriculture (Scott 2018).

10 Bonobos are not the peaceful species sometimes depicted in the media, but they do differ from chimpanzees in their aggressive behaviour: they do not sexually coerce females, do not commit infanticide and do not form lethal raids or border patrols (Hare et al. 2012; Tan et al. 2017).
